# Spatial mapping of PUFA incorporation into phospholipids with bisallylic deuteration

**DOI:** 10.1016/j.jlr.2025.100971

**Published:** 2025-12-31

**Authors:** Junji Obi, Kuniyuki Kano, Yuta Shimanaka, Shukichi Haisa, Nozomu Kono, Junken Aoki

**Affiliations:** Laboratory of Health Chemistry, Graduate School of Pharmaceutical Sciences, University of Tokyo, Bunkyo-ku, Tokyo, Japan

**Keywords:** MS imaging, bisallylic deuteration, PUFAs, dihomo-γ-linolenic acid, phospholipids

## Abstract

MS imaging using stable isotope-labeled fatty acids provides a groundbreaking approach to precisely localizing exogenous fatty acids and their metabolites in vivo. However, challenges persist, particularly with fatty acids labeled with fixed isotopic numbers, which can lead to spectral interferences and limit the number of metabolites that can be detected. In this study, we employed a bisallylic deuteration method to synthesize dihomo-γ-linolenic acid (DGLA) isotopes with *m/z* values ranging from +4 to +8 Da relative to endogenous DGLA, which allowed us to meticulously dissect DGLA metabolism in mice using LC-quadrupole TOF-MS and MS imaging. Our strategy enabled the clear selection of *m/z* values for phospholipids enriched with deuterated DGLA (D-DGLA) and deuterated arachidonic acid derived from D-DGLA, all while maintaining low background noise. This precision facilitated the successful visualization of D-DGLA and D-arachidonic acid-containing phospholipids in lung tissue, revealing their distinct localization compared with endogenous phospholipids. Our findings highlight bisallylic deuteration as a powerful tool for elucidating the in vivo dynamics of exogenous PUFAs through MS imaging techniques.

## Graphical Abstract

**Figure:**
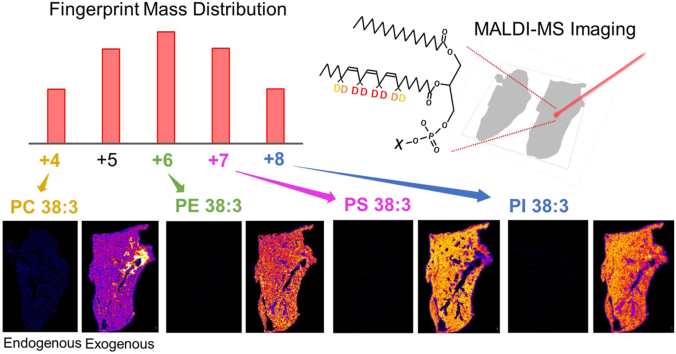

PUFAs, characterized by their multiple double bonds, are vital components of our diet and are classified into n-3 (ω3) and n-6 (ω6) series based on the position of their first double bond from the omega end (1). These essential fatty acids must be obtained through dietary sources, as they play crucial roles in maintaining our health. n-3 PUFAs, such as EPA and DHA, are abundant in fish and flaxseed oil, and are renowned for their anti-inflammatory properties. In contrast, n-6 PUFAs, including arachidonic acid (ARA), are prevalent in vegetable oils and nuts, and while essential, excessive intake can exacerbate inflammation. The intricate balance between n-6 and n-3 PUFAs is pivotal in modulating inflammatory pathways. ARA is a precursor to proinflammatory mediators like prostaglandins and leukotrienes (1, 2), whereas n-3 PUFAs are transformed into anti-inflammatory compounds (2). This dynamic interplay influences the development of inflammatory diseases, positioning n-3 supplementation as a preventative strategy (3, 4, 5, 6). Moreover, PUFAs are integral to cell membrane function, yet the precise impact of their incorporation into phospholipid molecules remains largely unexplored.

Dihomo-γ-linolenic acid (DGLA), a lesser-known n-6 PUFA, serves as an intermediary in the conversion to ARA. Despite its intermediate status, DGLA exists in measurable concentrations and fluctuates under certain disease conditions. Notably, studies have shown that DGLA level decreases in response to ozone exposure in mice, leading to inflammatory damage (7), whereas supplementation with DGLA or its precursor, γ-linolenic acid, has been shown to alleviate symptoms of inflammatory diseases, such as atopic dermatitis, rheumatoid arthritis, and allergic rhinitis (8, 9, 10). These findings underscore the potential therapeutic benefits of DGLA supplementation and suggest a need to re-evaluate the current focus on n-3 fatty acids in treating inflammatory diseases (11). Given the dual capacity of DGLA to be converted into both proinflammatory and anti-inflammatory agents, understanding the regulatory mechanisms governing this duality is essential for harnessing the full potential of DGLA as a dietary supplement.

The pharmacological actions of DGLA remain an intriguing area of study, with many aspects yet to be fully elucidated. Unlike ARA, DGLA is metabolized into potent anti-inflammatory mediators, including prostaglandin E1 and 15-hydroxyeicosatrienoic acid (12). For DGLA to be converted to these beneficial lipid mediators, it must first be incorporated into phospholipids and subsequently released as free fatty acids through phospholipase A_2_-mediated hydrolysis (13). Moreover, DGLA may serve a vital role as a structural component within cell membranes, embedded in phospholipid molecules. Elucidating the specific phospholipid species that incorporate DGLA and their cellular distribution in tissues are crucial for comprehending the functional roles of DGLA. Previous studies have focused on measuring DGLA levels in total lipids or total phospholipids within tissues; however, the detailed cellular distribution of DGLA and its phospholipid derivatives in specific tissues remains largely unexplored. This knowledge gap presents a compelling opportunity for further research to uncover the intricate dynamics of DGLA in cellular metabolism and its potential therapeutic applications.

MALDI-MS imaging is a powerful tool for visualizing the spatial distribution of metabolites, including phospholipids, within tissue sections (14). The application of MS imaging to phospholipid research has unveiled the spatial and differential distribution of a wide array of phospholipid species, each with unique headgroups and acyl chains (15). Furthermore, the use of stable isotope-labeled fatty acids enables us to elucidate the in vivo dynamics of exogenous fatty acids. However, several challenges remain for MS imaging. Because MALDI-MS imaging does not involve a separation step like LC-MS/MS, isobaric species often coexist and are codetected within a pixel, increasing spectral complexity and background. Conventional studies have used single-isotope-labeled fatty acids prepared by methods such as α-deuteration (16) and α,β-deuteration (17). A primary concern is the potential overlap in exact mass between labeled fatty acids and endogenous species, owing to the diversity of fatty acid structures and the presence of natural isotopes. This overlap elevates background, complicating the selective detection of labeled phospholipids.

A more robust solution involves using isotopologues, a set of stable isotope-labeled fatty acids with varied masses, enabling the selection of *m/z* values with minimal background interference. Bisallylic deuteration is a fatty acid-labeling method that enables us to replace the hydrogen with deuterium atoms selectively at the bisallylic methylene, the CH_2_ group flanked by two C=C double bonds in a PUFA (18). Since the replacement reactions proceed across multiple double bonds at varying efficiencies, unlike α-deuteration (16) and α,β-deuteration (17) methods, bisallylic deuteration gives rise to fatty acid isotopologues that have a different number of deuterium substitutions, that is, different molecular weights. In this study, we applied the bisallylic deuteration to DGLA, successfully synthesizing deuterated DGLA (D-DGLA) as five major isotopologues with mass shifts ranging from +4 to +8. Using the DGLA isotopologues, we could detect and map major D-DGLA-derived phospholipids by MS imaging, which was found to be difficult with other labeling strategies. Our findings also revealed that exogenous DGLA was preferentially incorporated into specific phospholipid species in defined regions of the lung, demonstrating the utility of this approach for studying lipid metabolism.

## Materials and methods

### Reagents

DGLA ethyl ester (DGLA-EE) was prepared from oil derived from *Mortierella alpina* (triglyceride form; DGLA accounting for 30% of total fatty acids) (19). The oil underwent alkaline-catalyzed ethyl esterification, and the resulting fatty acid ethyl esters were fractionated by precision distillation based on differences in vapor pressure (reflecting carbon chain length). The DGLA-enriched fraction was then further separated and purified by LC according to differences in the degree of unsaturation, yielding DGLA-EE with a purity exceeding 96% (20). Methanol (LC-MS grade), acetonitrile (LC-MS grade), and ethanol (HPLC grade) were purchased from Kanto Chemical Co, Inc (Tokyo, Japan). Methylcellulose solution (0.5 w/v%), acetone (super dehydrated), deuterium oxide, 2 M hydrochloric acid, 10% silver nitrate-impregnated silica gel, and 1 M ammonium formate solution were purchased from Fujifilm Wako Pure Chemical Corp (Osaka, Japan). *Para*-nitroaniline (*p*-NA) and Tris(acetonitrile)cyclopentadienylruthenium(II) hexafluorophosphate (Ru catalyst) were purchased from Sigma-Aldrich (St Louis, MO).

### Synthesis of D-DGLA

A modified procedure based on a previously reported method was employed (18). Briefly, 800 mg of DGLA-EE was accurately weighed into a round-bottom flask equipped with a magnetic stirrer and a side arm. Deuterium oxide (10 ml) and 70 ml of acetone were added, and the mixture was stirred to dissolve DGLA-EE. Separately, 60 mg of Ru catalyst (15 wt% relative to DGLA-EE) was weighed into a glass vial and dissolved in 10 ml of acetone. The catalyst solution was then added to the DGLA-EE solution, and the mixture was stirred at room temperature for 1 h. All steps were carried out under an argon atmosphere. Prior to use, all solvents were degassed by bubbling with argon. The reaction was quenched by the addition of 10 ml of 2 N hydrochloric acid. The resulting mixture was transferred to a separatory funnel and subjected to liquid-liquid extraction with 200 ml of *n*-hexane and 200 ml of water. After phase separation, the organic layer was collected. The extraction with 200 ml of *n*-hexane was repeated three times. The combined organic layers were concentrated using a rotary evaporator to afford the crude D-DGLA. Crude D-DGLA was further purified by column chromatography using 10% silver nitrate-impregnated silica gel to remove residual impurities. The mass distribution of synthesized D-DGLA and DGLA-EE was analyzed using an Agilent 7890A GC system (Agilent Technologies, Palo Alto, CA) coupled with a quadrupole TOF mass spectrometer (Xevo G2-XS QTof; Waters Corporation, Manchester, UK) equipped with an atmospheric pressure GC ion source (20). The purified D-DGLA was stored under an argon atmosphere at −30°C.

### Animal experiments

Seven-week-old male C57BL/6JJcl mice (CLEA Japan, Inc, Tokyo, Japan) were housed in plastic cages under standard conditions with ad libitum access to tap water and a standard diet (CE-2; CLEA Japan, Inc). After a 7-day acclimatization period, mice were administered D-DGLA via oral gavage once daily for 7 consecutive days at a dose of 1,000 mg/kg body weight. D-DGLA was suspended in a 0.5% (w/v) methylcellulose solution to a final concentration of 5% (w/v). Control animals received only the 0.5% (w/v) methylcellulose vehicle. All animal experiments were conducted in accordance with the Standards Relating to the Care and Management of Experimental Animals in Japan and were approved by the Animal Care and Use Committee of the University of Tokyo.

### Tissue collection and section preparation

Mice were anesthetized on the day following the final administration of D-DGLA. After excision of the right atrium, perfusion was performed with PBS. For LC-quadrupole TOF MS (LC-QTof-MS) analysis, the lungs were excised, weighed, and homogenized in methanol to achieve a final concentration of 40 mg tissue/ml. Tissue homogenization was carried out using a Micro Smash homogenizer equipped with zirconia beads (TOMY, Tokyo, Japan) at 3,500 rpm for 60 s. The homogenate was centrifuged at 20,000*g* for 5 min, and the resulting supernatant was collected for analysis. For MALDI-MS imaging, the lungs were embedded in 2% carboxymethyl cellulose using liquid nitrogen. The embedded tissues were cryosectioned at a thickness of 15 μm and then thaw-mounted onto MAS-coated glass slides (Matsunami Glass Ind, Osaka, Japan). All samples were stored at −80°C until analysis.

### LC-QTof-MS

LC-QToF-MS analysis was performed using an ultraperformance LC system (ACQUITY UPLC; Waters Corporation, Milford, MA) coupled to a quadrupole TOF mass spectrometer (Cyclic IMS; Waters Corporation, Milford, MA) with an electrospray ionization source. Data were acquired in full-scan mode over an *m/z* range of 400–1,000 with a mass resolution of 70,000. Chromatographic separation was achieved using a reversed-phase column (L-column3 C18 column, 2.0 mm i.d. × 100 mm length, 2 μm particle size; CERI, Tokyo, Japan) maintained at 45°C. The mobile phases consisted of (A) acetonitrile/water (3:2, v/v) containing 10 mM ammonium formate and (B) isopropanol/acetonitrile (9:1, v/v) containing 10 mM ammonium formate. The flow rate was set to 0.3 ml/min. A linear gradient was applied as follows: 0–2 min, 5% B; 2–26.4 min, 5% B to 100% B; 26.4–36 min, 100% B; 36–36.1 min, 5% B; and 36.1–40 min, 5% B for re-equilibration (21). The capillary voltage, sampling cone voltage, and desolvation temperature were set to 2.5 kV, 30 V, and 450°C, respectively. For endogenous lipid species, product ion (MS/MS) spectra were acquired, and the fatty acyl compositions were assigned based on the characteristic fragment ions. For the labeled species, peaks were annotated based on the retention times corresponding to the respective unlabeled lipids.

### MALDI-MS imaging

MALDI-MS imaging was performed according to previously reported methods (22, 23). Briefly, tissue sections were thaw-mounted and air-dried, followed by a 5-s wash in 50 mM ammonium formate. *p*-NA was dissolved in 80% ethanol containing 150 mM ammonium formate. The solution was sprayed onto the sections using a SunCollect automatic sprayer (SunChrom, Germany) at a pressure of 0.3 MPa and a flow rate of 15 μl/min. Lung sections were coated with 10 layers of 1 mg/ml *p*-NA, followed by 25 layers of 10 mg/ml *p*-NA. MALDI-MS imaging was performed using a MALDI laser system (AP-SMALDI5; TransMIT GmbH, Giessen, Germany) coupled to a Fourier transform orbitrap mass spectrometer (Q Exactive; Thermo Fisher Scientific, San Jose, CA). Data were acquired in full-scan mode over an *m/z* range of 700–950 at a mass resolution of 140,000 (at *m/z* = 200). Spray voltage, capillary temperature, and S-lens RF level were set to 4.0 kV, 400°C, and 100 (instrument units), respectively. The AP-SMALDI5 system was equipped with a solid-state UV laser, which was manually focused. Laser irradiation was performed at 70 μm intervals and was controlled using an attenuator set to 28°. Ion images were reconstructed using IMAGEREVEAL MS (Shimadzu Corporation, Kyoto, Japan) with a mass tolerance of ±5 ppm. The analytes detected by both LC-QTof-MS analysis and MALDI-MS imaging are summarized in Supplemental Table S1.

## Results

**Fig. 1:**
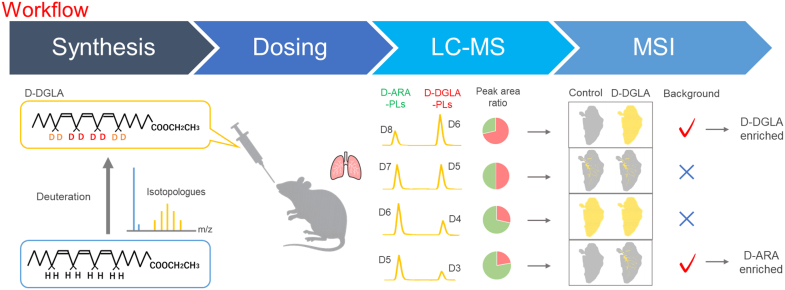
Overall workflow of this study. Synthesis: D-DGLA was synthesized as a series of isotopologues with different numbers of deuterium atoms, and their deuterium substitution patterns were evaluated. Dosing: Mice were orally administered D-DGLA, and lung tissues were collected after dosing. LC-MS: Incorporation of D-DGLA and its metabolite D-ARA into pulmonary phospholipids was quantified using LC-QTof-MS and calculating peak area ratios. MSI: MS imaging was performed on lung sections to visualize the spatial distribution of D-DGLA- and D-ARA-containing phospholipids. Combinations of phospholipid classes and D-DGLA isotopologues were compared to identify conditions with low endogenous background signals and improved detection of exogenous labeled species.

A schematic of the experimental workflow is shown in Fig. 1 to provide an overview of the study design. We first synthesized D-DGLA as a series of isotopologues and examined their deuterium substitution rate. Mice were then orally administered D-DGLA, and lung tissues were collected. The uptake of D-DGLA into pulmonary phospholipids was evaluated using both LC-QTof-MS and MS imaging. In MS imaging analysis, endogenous background noise often interferes with the detection of exogenously added labeled compounds and their derivatives. Therefore, we investigated combinations of phospholipids and D-DGLA isotopologues with low background noise. The uptake of D-ARA, converted from D-DGLA, into phospholipids was similarly evaluated using LC-QTof-MS and MS imaging.

### Synthesis of D-DGLA isotopologues by a bisallylic deuteration method

**Fig. 2:**
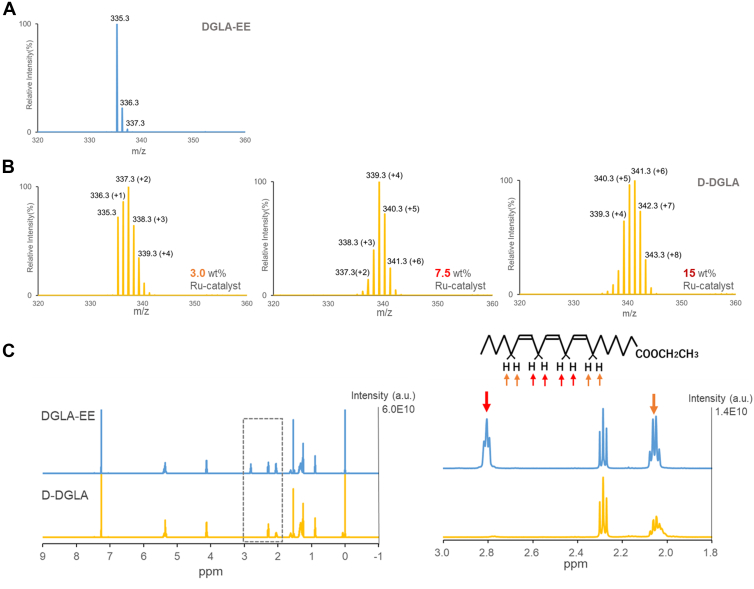
Evaluation of the synthesized D-DGLA used in this study. A: Mass spectrum of DGLA-EE, the starting material. B: Mass spectra of D-DGLA synthesized using Ru catalysts at 3.0, 7.5, and 15 wt% of the starting material (DGLA-EE). Note that deuterium incorporation into D-DGLA increases with the amount of Ru catalyst used. C: 1H NMR spectra of DGLA-EE and D-DGLA (synthesized using 15 wt% Ru catalyst). The signal intensities at 2.8 and 2.0 ppm correspond to the bisallylic and monoallylic protons, respectively. These data indicate that, upon the reaction, most of the bisallylic protons and a portion of the monoallylic protons were replaced by deuterium. The signal intensity around 2.3 ppm arises from the α-protons and is not affected by this reaction.

We utilized the bisallylic deuteration method to generate a series of D-DGLA isotopologues. In GC-QTof-MS analysis, unlabeled DGLA-EE, the starting material, showed a significant peak at *m/z* 335.3 and minor peaks at *m/z* 336.3 and *m/z* 337.3 derived from natural isotopes (Fig. 2A). Deuterium incorporation increased with concentrations of a Ru catalyst (Fig. 2B). Using 15% (w/w) of the catalyst, we obtained a mixture of D-DGLA isotopologues with a specific “fingerprint” mass distribution ranging from *m/z* 339.3 (+4) to 343.3 (+8) (Fig. 2B). Notably, this mass range (+4 to +8) has not been utilized in previous in vivo PUFA administration studies for MS imaging applications. The mass distribution of D-DGLA isotopologues centered at 341.3 (+6). To determine whether deuterated atoms are preferentially incorporated at the bisallylic position or monoallylic position, we performed 1H NMR analysis (Fig. 2C). As expected, the D-DGLA isotopologues (+4 to +8) showed almost complete deuteration of the four bisallylic hydrogen atoms, along with partial substitution at the monoallylic position. Of note, no appreciable changes were observed for protons at other positions, including the α-positions (Fig. 2C).

### Phospholipid species incorporating D-DGLA and D-ARA in the lung

**Fig. 3:**
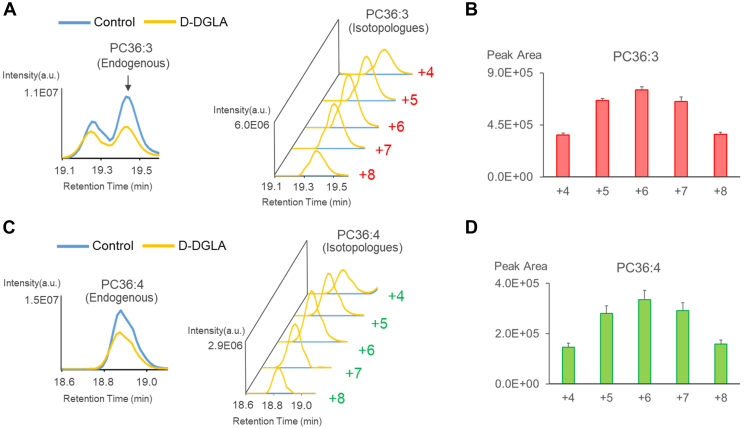
Analysis of D-DGLA-derived phospholipids in the mouse lung administered with D-DGLA by LC-QTof-MS. A: Extracted ion chromatograms for endogenous PC 36:3 (*left*) and the corresponding PC 36:3 isotopologues (*right*) in the lung of mice fed with a control diet (*blue*) and mice administered with D-DGLA (*yellow*). Note that the peak for endogenous PC 36:3 eluting at 19.2 min is expected to be a composition isomer (PC 18:1_18:2). B: Peak areas of PC 36:3 isotopologues (A, *right*) in the lung of D-DGLA-administered mice. Data are presented as mean + SD (n = 3 biological replicates; individual mice). C: Extracted ion chromatograms for endogenous PC 36:4 (*left*) and the corresponding PC 36:4 isotopologues (*right*) in the lung of mice fed with a control diet (*blue*) and mice administered with D-DGLA (*yellow*). D: Peak areas of PC 36:4 isotopologues (C, *right*) in the lung of D-DGLA-administered mice. Data are presented as mean + SD (n = 3 biological replicates; individual mice). Results for other analytes are shown in Supplemental Figures S2 and S3. “+X” indicates the nominal mass shift (dalton) of each D-DGLA isotopologue relative to unlabeled DGLA (defined as +0), corresponding to the number of incorporated deuterium atoms.

We attempted to identify the phospholipid species that incorporated exogenous DGLA. First, we confirmed that various classes of DGLA-containing phospholipids, including phosphatidylcholine (PC), phosphatidylethanolamine (PE), phosphatidylinositol (PI), and phosphatidylserine (PS), increased in the lung when mice were orally administered unlabeled DGLA-EE at 1,000 mg/kg for 7 days (Supplemental Fig. S1A). This dose falls within the range used in previous supplementation studies with PUFAs, such as DHA and EPA (24, 25, 26). At this dose, we observed neither obvious changes in appearance or behavior nor any effects on body weight in mice (Ctrl: 22.9 ± 1.4 g vs. D-DGLA: 23.1 ± 1.2 g). The major phospholipid species incorporating DGLA included PE 38:3 (18:0_20:3), PE 36:3 (16:0_20:3), PE O-36:4 (P-16:0_20:3), PE O-38:4 (P-18:0_20:3), PC 38:3 (18:0_20:3), PC 36:3 (16:0_20:3), PS 38:3 (18:0_20:3), PI 38:3 (18:0_20:3), and PI 36:3 (16:0_20:3) (Supplemental Fig. S1B). These phospholipid species in the lung also incorporated D-DGLA isotopologues when mice were administered D-DGLA isotopologues at 1,000 mg/kg for 7 days (the same dosage as unlabeled DGLA-EE) (Fig. 3A, Supplemental Fig. S2A). The phospholipid species exhibited a characteristic “fingerprint” mass distribution ranging from +4 to +8, with a maximum signal intensity centered at +6 (Fig. 3B, Supplemental Fig. S2B), as observed for D-DGLA (Fig. 2B). These phospholipid species incorporating D-DGLA were not detected in the lungs of control mice (Fig. 3A, Supplemental Fig. S2A). The proportion of D-DGLA versus endogenous DGLA in each DGLA-containing phospholipid ranged from 65.1% to 81.4% (Supplemental Table S2), suggesting uniform incorporation of D-DGLA isotopologues into lung phospholipids, regardless of the head groups and the paired fatty acids.

D-DGLA is converted to D-ARA through a Δ-5 desaturase and incorporated into phospholipids in vivo (12). In fact, LC-QTof-MS analysis of the lung phospholipids detected various D-ARA-containing phospholipids, including PC 36:4 (16:0_20:4), PC 38:4 (18:0_20:4), PE 36:4 (16:0_20:4), PE 38:4 (18:0_20:4), PE O-36:5 (P-16:0_20:4), PE O-38:5 (P-18:0_20:4), PS 38:4 (18:0_20:4), PI 36:4 (16:0_20:4), and PI 38:4 (18:0_20:4), which also displayed a fingerprint mass distribution similar to that of D-DGLA (Fig. 3C, D, Supplemental Fig. S3). Based on these results, we set nine D-DGLA-containing (PE 38:3, PE 36:3, PE O36:4, PE O38:4, PC 38:3, PC 36:3, PS 38:3, PI 38:3, and PI 36:3) and nine D-ARA-containing (PC 36:4, PC 38:4, PE 36:4, PE 38:4, PE O-36:5, PE O-38:5, PS 38:4, PI 36:4, and PI 38:4) phospholipids as targets for the following MS imaging analyses.

### Selecting *m/z* with minimal background noise for MALDI-MS imaging

**Fig. 4:**
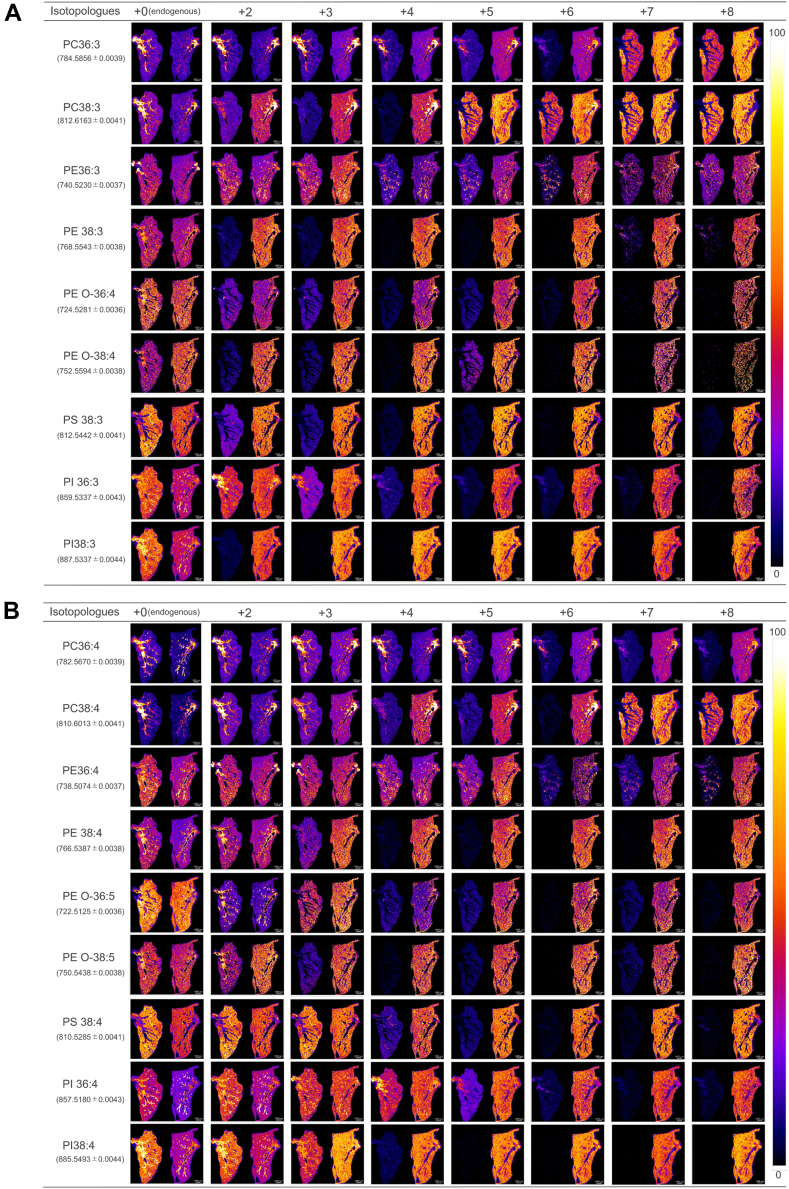
MALDI-MS image at selected *m/z* for isotopologues of (A) D-DGLA- and (B) D-ARA-containing phospholipids. In each panel, the left sections are from the control mouse and the right sections are from the mouse-administered D-DGLA. *m/z* values for each endogenous phospholipid are shown in the left parentheses. “+X” indicates the nominal mass shift (dalton) of each D-DGLA isotopologue relative to unlabeled DGLA (defined as +0), corresponding to the number of incorporated deuterium atoms. The MS imaging experiment was independently performed twice with similar results, and representative images are shown. Note that strong signals were detected in the control sections for several isotopologues, reflecting background noise.

To accurately determine the spatial distribution of phospholipids incorporating D-DGLA, it is essential to select *m/z* values with minimal background interference. We obtained MALDI-MS imaging data of the above-mentioned 18 target phospholipids (nine D-DGLA-containing and D-ARA-containing phospholipids) in the range from +2 to +8 in the lung sections from both mice administered D-DGLA and control mice (Fig. 4). This analysis highlighted the importance of selecting appropriate isotopologues for the detection of phospholipid species in MS imaging. For example, in the case of PC 36:3, the signal for the +2 isotopologue was detected to the same extent in both D-DGLA-administered and control lung sections (Fig. 4A), indicating that the +2 signal is a false signal. In the case of PE 38:3, isotopologues +4, +5, and +6 achieved clear low background noise. Background noises were relatively high for +2 and +3 isotopologues in several phospholipid species (Fig. 4), possibly due to the naturally occurring isotopes, such as ^13^C. This result also suggests that the previously used +2 (16) and +3 (27) strategies for PUFA labeling are not always suitable. Importantly, we were able to select at least one isotopologue for each phospholipid species with weak or almost no detectable background signals (Fig. 4). These results clearly indicate that MS imaging of phospholipids relying on a single isotopic fatty acid signal is not always suitable and highlight the usefulness of isotopic fatty acids containing multiple isotopologues.

### Discrimination between D-DGLA- and D-ARA-containing phospholipids

**Fig. 5:**
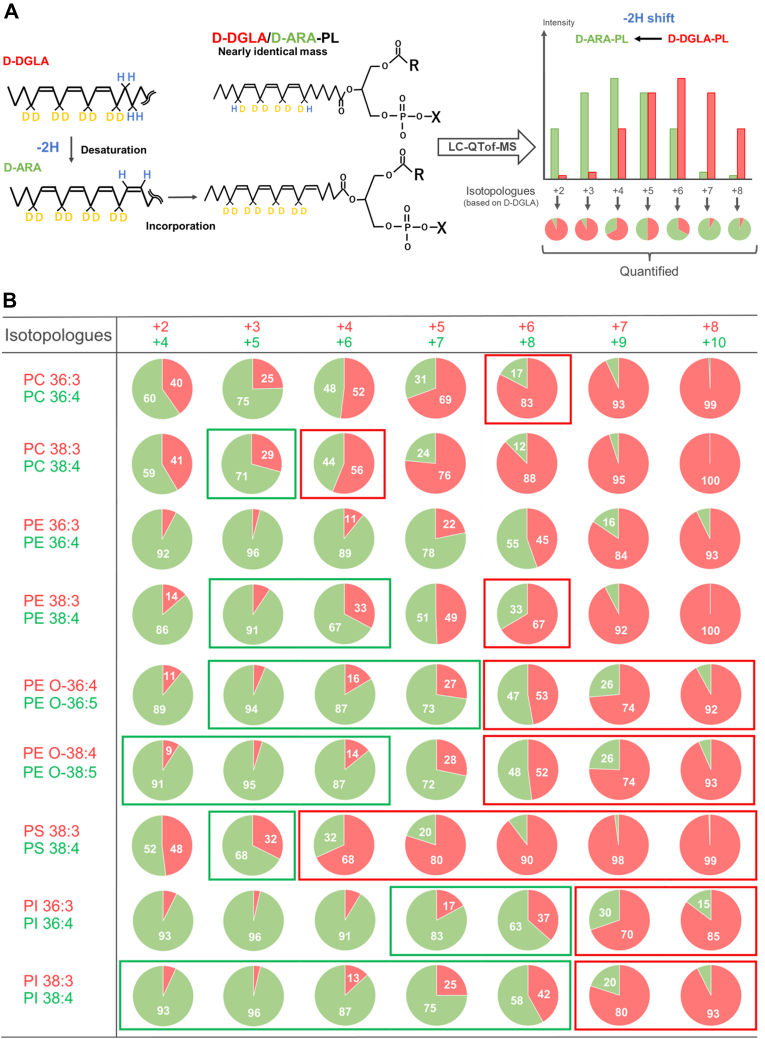
D-DGLA/D-ARA ratios of phospholipids (PLs) with nearly identical *m/z* values. A: Evaluation strategy for determining the ratio of D-DGLA- and D-ARA-containing PLs. D-DGLA can be converted to D-ARA, which is also incorporated into PLs. The *m/z* values of PLs containing D-DGLA are nearly equal to those of PLs containing D-ARA with two additional deuterium atoms compared with D-DGLA. These D-DGLA- and D-ARA-containing PLs with close *m/z* values can be resolved and quantified separately by LC-QToF-MS, enabling us to determine their ratios. B: Relative amounts of D-DGLA- and D-ARA-containing PLs with close *m/z* values were separated by LC-QTof-MS and quantified for nine PL species in four PL classes. “+X” denotes the nominal mass shift (dalton) of each D-DGLA isotopologue relative to unlabeled DGLA (defined as +0), corresponding to the number of incorporated deuterium atoms. Boxes indicate pairs of D-DGLA- and D-ARA-containing PLs with low background noise in MS imaging. *Red* and *green* boxes indicate pairs with higher signal intensities for D-DGLA- and D-ARA-containing PLs, respectively.

D-DGLA and D-ARA differ in one double bond, that is, they differ in two hydrogen atoms, so the *m/z* values of their isotopologues can overlap. For example, PE 38:3 with six deuterium atoms (+6) and PE 38:4 with eight deuterium atoms (+8) have nearly identical masses (774.5920 vs. 774.5889), making them difficult to distinguish in MS imaging analysis using conventional mass resolution (<300,000) (Supplemental Fig. S4). To distinguish between D-DGLA- and D-ARA-containing phospholipids by MALDI MS imaging, it is essential to select *m/z* values at which one signal intensity is dominant over the other. Thus, based on the LC-QTof-MS results, which enable us to detect D-DGLA- and D-ARA-phospholipids separately, we quantified the relative abundance of D-DGLA- versus D-ARA-containing phospholipids for each *m/z* value (Fig. 5A). For PC and PS, selecting isotopologues with +4 or higher *m/z* values ensured that D-DGLA signals comprised at least 50% of total intensity (Fig. 5B). In contrast, for PE and PI, in which ARA was more readily incorporated, selecting +7 or +8 isotopologues allows detection of D-DGLA-predominant species (Fig. 5B).

By combining the results of lower background noise (Fig. 4) and biased ratio of D-DGLA- and D-ARA-containing phospholipids (Fig. 5A), PI with 38 carbons showed >90% contribution of D-ARA for PI 38:4 (+5) and of D-DGLA for PI 38:3 (+8) (Fig. 5B), enabling each species to be visualized with high specificity. Although complete discrimination could not be achieved in all cases, we identified *m/z* values with high contributions of either D-DGLA-containing phospholipid or D-ARA-containing phospholipid for 15 of the 18 phospholipid species in lung sections. In Fig. 5B, the green boxes show that D-DGLA-containing phospholipids were predominant, whereas the red boxes containing D-ARA-phospholipids were predominant, both with low endogenous background. In our present condition, only three species, PC 36:4, PE 36:3, and PE 36:4, could not be fully resolved.

### Localization of D-DGLA-derived phospholipids in the lung in mice

**Fig. 6:**
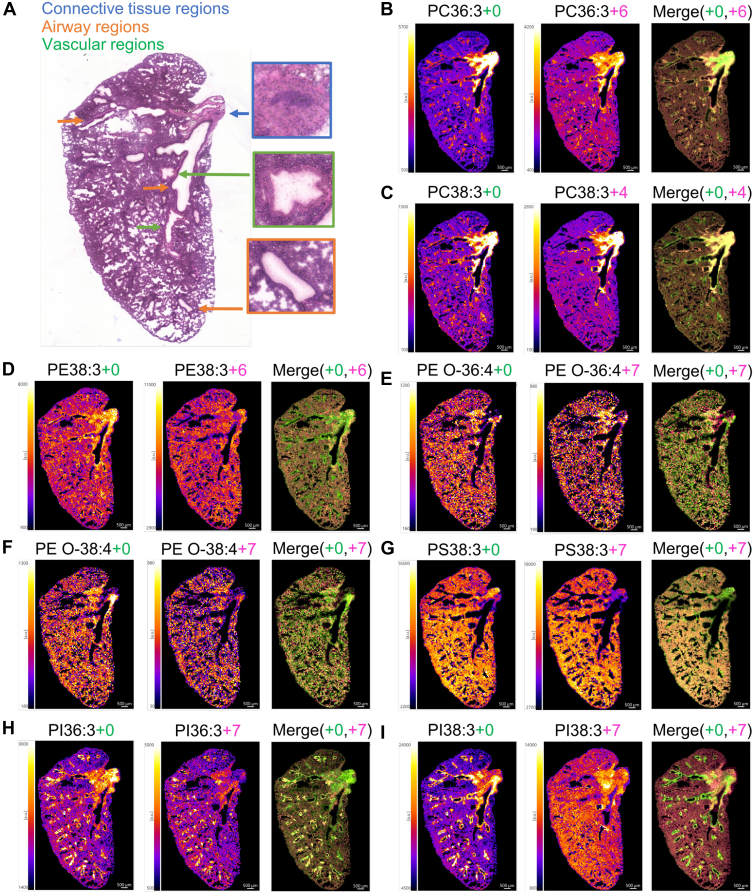
Visualization of D-DGLA-containing phospholipids (PLs) in the mouse lung in comparison with PLs containing endogenous DGLA. A: H&E staining of a lung section. B–I: In each panel, the distribution of endogenous DGLA-containing PL (PL+0) is shown on the left, D-DGLA-containing PL (PL + X) is in the middle, and the merged image is on the right. Isotopologues with the lowest background, shown in Figure 5, were selected. The isotopologues selected are (B) PC 36:3 + 6, (C) PC 38:3 + 4, (D) PE 38:3 + 6, (E) PE O-36:4 + 7, (F) PE O-38:4 + 7, (G) PS 38:3 + 7, (H) PI 36:3 + 7, and (I) PI 38:3 + 7. Spatial resolution = 70 μm. “+X” denotes the nominal mass shift (dalton) of each D-DGLA isotopologue relative to unlabeled DGLA (defined as +0), corresponding to the number of incorporated deuterium atoms. The MS imaging experiment was independently performed twice with similar results, and representative images are shown. In the merged images, signals from endogenous lipids are shown in *green*, and those from D-DGLA-containing lipids are shown in magenta.

MALDI-MS imaging successfully visualized the localization of D-DGLA- and D-ARA-containing phospholipids in alveolar, airway, vascular, and connective tissue regions (Fig. 6 and Supplemental Fig. S5). For PC 36:3, PE O-36:4, PE O-38:4, and PS 38:3, exogenous DGLA was incorporated in patterns consistent with endogenous species: connective tissue and airways for PC 36:3, and throughout the lung for PE O-36:4, PE O-38:4, and PS 38:3 (Fig. 6B, E, F, G). Notably, in some cases, the spatial distribution of exogenous D-DGLA-containing PLs differed from their endogenous counterparts. For instance, endogenous PC 38:3 was predominantly observed in the connective tissue, airway, and vascular regions, whereas PC 38:3-incorporated exogenous D-DGLA was observed mainly in the connective tissue but not in the airway regions (Fig. 6C, Supplemental Fig. S6). Similarly, although endogenous PE 38:3 and PI 38:3 localized to airway regions, their D-DGLA-containing forms were not appreciably detected in these regions (Fig. 6D, I). In contrast, both endogenous and exogenous PI 36:3 species were clearly distributed within the airway region (Fig. 6H).

For D-ARA-containing phospholipids, as with D-DGLA, we identified species that exhibited localization similar to their endogenous counterparts (e.g., PE O-38:5, PS 38:4, PI 36:4) as well as species with distinct distributions (e.g., PC 38:4, PE O-36:5, PI 38:4, PE 38:4) (Supplemental Fig. S5). Importantly, focusing on the in vivo disposition of exogenous DGLA and ARA, we found that structurally similar phospholipid species (e.g., PC 38:3 and PC 38:4) exhibited nearly identical spatial distributions (Supplemental Fig. S7).

## Discussion

In this study, we represented a novel method for spatially mapping phospholipid molecules incorporating exogenously administered DGLA in mice by MALDI-MS imaging combined with bisallylic deuterium labeling. The synthesized bisallylic-D-DGLA, which exhibited a specific fingerprint mass distribution pattern ranging from +4 to +8, reduced the background interference from endogenous molecules by enabling the selection of optimal *m/z* values. The present method has several advantages over existing methods.

First, the present method enabled us to localize numerous D-DGLA-derived phospholipids on tissue sections. Indeed, we successfully visualized eight phospholipid species incorporating D-DGLA (Fig. 5). In this study, we also succeeded in mapping seven phospholipids that incorporate the ARA converted from DGLA (Fig. 5). Using homogenously labeled fatty acids with molecular weights +2 or +3, Yoshinaga *et al.* (16, 27) evaluated the uptake of DHA or ARA into phospholipids in zebrafish or mice, and they were able to visualize two or three PC species, respectively.

In addition to its characteristics to detect numerous phospholipid species, the present method required only a smaller amount of isotopically labeled fatty acids. In the earlier work by Yoshinaga *et al.* (16) evaluating DHA incorporation into phospholipids, they administered a cumulative dose of 2.7 mmol of a homogeneous +2 isotopologue to juvenile mice. In the present study, by contrast, we administered only 0.5 mmol of D-DGLA (1,000 mg/kg for 7 days) and were able to detect multiple phospholipid species. It should be emphasized here that MALDI-MS imaging was still successfully achieved despite the fact that our isotope was a mixture of multiple isotopologues (+4 to +8), resulting in even lower quantities of individual isotopologues.

This analysis focused on the deuterated form of DGLA, but this method is applicable to various PUFAs. Analysis of background noise in control lung sections revealed that this method enables optimal *m/z* selection, even for phospholipids containing other PUFAs, such as DHA. Stronger background signals were observed for putative isotopologues of DHA-containing phospholipids ranging from +2 to +5 (Supplemental Fig. S8), indicating that homogeneous +2, +3, or +4 isotopologues for DHA, commonly used in earlier studies, may not be ideal as MS imaging probes. In contrast, the bisallylic deuteration is expected to yield D-DHA isotopologues ranging from +8 to +12. PC 38:6 showed minimal background at +8 and +10, and PC 40:6 exhibits a minimum at +8 (Supplemental Fig. S8). Other DHA-PLs, including PE 38:6, PE 40:6, PS 38:6, PS 40:6, PI 38:6, and PI 40:6, were predicted to have low background signals ranging from +8 to +14 (Supplemental Fig. S8). Thus, bisallylic deuteration appears broadly effective for MS imaging of phospholipids containing variously labeled PUFAs.

Our approach has identified lung regions where D-DGLA and D-ARA are preferentially incorporated into phospholipids. For example, our MALDI-MS imaging analysis revealed that D-DGLA is preferentially incorporated into PC 36:3 and PI 36:3 in the airway region. Interestingly, Li *et al.* (7) reported that supplementation with DGLA attenuates ozone-induced airway inflammation in mouse models of lung injury. Thus, the preferential D-DGLA incorporation in the airway region nicely explains the pharmacological action of DGLA to alleviate the airway inflammation.

The molecular mechanism by which DGLA is incorporated into phospholipids remains unknown. Interestingly, DGLA-containing PC species markedly decreased in the liver of *Lpcat3* (*Lplat12*) knockout mice (28). LPCAT3 is a lysophospholipid acyltransferase that incorporates mainly ARA into phospholipids. Considering the structural similarity between DGLA and ARA, it is reasonable to assume that LPCAT3 is one of the enzymes responsible for incorporating DGLA into phospholipids via the remodeling pathway. The combined use of specific enzyme inhibitors or knockout mice with the present method will help to dissect the mechanisms by which D-DGLA is incorporated into phospholipids and to clarify how these processes relate to its pharmacological actions.

In specific regions in the lung, we observed differential distributions of D-DGLA- or D-ARA-containing phospholipids from those of their endogenous counterparts. For example, endogenous PI 38:3 and PI 38:4 were predominantly localized to the bronchial epithelium, whereas newly formed labeled PIs were more enriched in the alveolar region (Fig. 6I and Supplemental Fig. S5G). Since DGLA content in the standard rodent chow is quite low, most endogenous DGLA is thought to be synthesized from linoleic acid, an n-6 fatty acid rich in the chow, through elongation and desaturation reactions and then converted to ARA. Exogenously administered DGLA bypasses these upstream metabolic pathways and is incorporated into phospholipids directly, which may partly explain the differential distributions. An alternative explanation is that newly synthesized PI 38:3 and PI 38:4 in the alveoli undergo progressive turnover or remodeling, ultimately resulting in an epithelial-dominant distribution at steady state. This scenario would imply the existence of as-yet-unidentified enzyme(s) responsible for the preferential degradation or remodeling of PI 38:3 and PI 38:4.

Several limitations in this study should be considered, including the incomplete resolution of (1) D-DGLA- and D-ARA-containing phospholipids, (2) structural isomers arising from different combinations of fatty acyl chains, and (3) isomeric 20:3 fatty acids themselves, such as DGLA (n-6) and mead acid (n-9). First, some D-DGLA- and D-ARA-containing species (e.g., D6-PC 38:3 and D8-PC 38:4) have overlapping *m/z* values at the mass resolution used in this study (140,000 at *m/z* 200), making them difficult to separate completely (Supplemental Fig. S4). To minimize such crosscontamination, we selected the most appropriate *m/z* values for each isotopologue based on LC-QTof-MS analysis (Fig. 5). However, it should be noted that LC-QTof-MS provides information on the average lipid composition of whole-tissue extracts, and local lipid ratios in specific regions may differ from this tissue average. A straightforward way to address this issue would be to employ higher-resolution mass analyzers, such as Fourier transform ion cyclotron resonance MS. Tiquet *et al.* (29) demonstrated using Fourier transform ion cyclotron resonance MS imaging that two species at nearly *m/z* 782.57 differing by Δ*m/z* = 0.0052 could be clearly separated (mass resolution = 400,000 at *m/z* 400), suggesting that higher mass resolution would substantially improve the separation of the two phospholipid species. Even with increased mass resolution, however, phospholipids with identical elemental compositions cannot be distinguished at the MS^1^ level. For example, the sum composition PC 36:3 is likely to correspond primarily to two major candidates, PC 16:0/20:3 and PC 18:1/18:2 (Fig. 3A). In fact, additional LC-MS/MS experiments showed that PC 38:4 is composed of multiple isomeric species (PC 18:1/20:3, PC 16:0/22:4, and PC 18:0/20:4) (Supplemental Fig. S9). Although we did not detect clear evidence of structural isomers for the 36:4 and 38:4 species in other phospholipid classes, potential isomeric contamination must still be considered when interpreting MS imaging data acquired in full-scan mode. In such situations, additional MS/MS measurements are required when a more detailed structural assignment is necessary. Another issue is the potential presence of isomers of DGLA. For 20:3 fatty acids, mead acid (20:3n-9) may coexist with DGLA (20:3n-6), and under the present conditions, we cannot entirely exclude its contribution to the measured signals. Ion mobility-MS has the potential to address these challenges. Using cyclic ion mobility, Poad *et al.* (30) showed that the Na^+^ adduct of PC 16:0/18:1 n-7 elutes earlier than the corresponding n-9 and n-10 isomers, enabling their separation. Nevertheless, irrespective of the analytical platform employed in future studies, the labeling strategy described here will retain the important advantage of allowing the selection of *m/z* values with minimal background signals.

Overall, our findings demonstrate that bisallylic deuteration, in combination with careful isotopologue selection and MALDI-MS imaging, offers a versatile and sensitive tool for mapping phospholipids incorporating exogenously added PUFA *in situ*. We anticipate that this strategy can be readily extended to other labeled fatty acids and organ systems, thereby facilitating a deeper understanding of how dietary and pharmacological PUFAs are handled at the tissue and cell-type levels.

## Data Availability

The datasets used and analyzed during the current study are available from the corresponding author on reasonable request.

## Supplemental Data

This article contains supplemental data.

## Conflict of Interest

The authors declare that they have no conflicts of interest with the contents of this article.
