# Supplementary material for: Spatial mapping of PUFA incorporation into phospholipids with bisallylic deuteration
Source: J Lipid Res. 2025 Dec 31;67(3):100971. doi: 10.1016/j.jlr.2025.100971 (PMC12955629; doi:10.1016/j.jlr.2025.100971)
Supplement: Supplemental Figures and Tables [file mmc1.pdf]

Supporting information

Spatial Mapping of PUFA Incorporation into Phospholipids with bisallylic deuteration

Authors
Junji Obi, Kuniyuki Kano*, Yuta Shimanaka, Shukichi Haisa, Nozomu Kono, Junken Aoki*

Affiliations
Department of Health Chemistry, Graduate School of Pharmaceutical Sciences, The University of Tokyo, Tokyo 113-0033, Japan

* Corresponding author; k-kano@mol.f.u-tokyo.ac.jp (K.K.), jaoki@mol.f.u-tokyo.ac.jp (J.A.)

Table of Content

Table/Figure	Title	Page
Supplementary Methods	Animal Experiments — Pre-study Using DGLA-EE<br>LC-MS/MS Analysis	S3
Table S1	Target analytes for LC-QTof-MS and MALDI-MS imaging	S4
Figure S1	Analysis of DGLA-containing phospholipids in the mouse lung by LC-MS/MS	S5
Figure S2	Analysis of D-DGLA-containing-phospholipids in the mouse lung administered with D-DGLA by LC-QTof-MS	S6
Figure S3	Analysis of D-ARA-containing-phospholipids in the mouse lung administered with D-DGLA by LC-QTof-MS	S7
Table S2	Substitution rate of DGLA and ARA-containing phospholipids	S8
Figure S4	An example of overlapping m/z values between D-DGLA- and D-ARA-containing phospholipids.	S9
Figure S5	Visualization of D-ARA-containing phospholipids in the mouse lung in comparison with ARA-containing phospholipids.	S10
Figure S6	D-DGLA is differently incorporated into PC36:3 and PC38:3	S11
Figure S7	Visualization of D-DGLA-containing phospholipids in the mouse lung in comparison with D-ARA-containing phospholipids.	S12
Figure S8	Comparison of relative intensities of m/z values putatively corresponding to DHA isotopologues in mouse lung by MALDI-MS imaging	S13
Figure S9	An example in which multiple D-DGLA-derived phospholipids with the same molecular formula were detected.	S14

Supplementary Methods

Animal Experiments — Pre-study Using DGLA-EE

The mice received DGLA-EE in instead of D-DGLA. All other parameters (Animal housing, acclimatization, and general handling) were identical in the Main Methods section (*Animal Experiments*).

LC-MS/MS Analysis

To profile DGLA-containing phospholipids, lipidomic analysis was performed using a Nexera X3 UHPLC system (Shimadzu, Kyoto, Japan) coupled to a QTRAP 7500 hybrid triple quadrupole linear ion trap mass spectrometer (SCIEX, Framingham, MA, USA) equipped with an electrospray ionization (ESI) source operated in negative ion mode. A 5 µL aliquot of the lipid extract, corresponding to 100 pmol of phosphorus, was injected via an autosampler. Chromatographic separation was achieved on a SeQuant ZIC-HILIC PEEK-coated column (250 mm × 2.1 mm, 3.5 µm; Merck, Darmstadt, Germany) maintained at 50 °C. The mobile phases consisted of (A) water/acetonitrile (50:50, v/v) containing 20 mM ammonium acetate, and (B) water/acetonitrile (95:5, v/v) containing 10 mM ammonium acetate. A linear gradient was applied as follows: 0–11 min, 100% B to 48% B; 11–15 min, 48% B to 10% B; 15–15.5 min, 10% B; 15.5–15.51 min, 10% B to 100% B; and 15.51–20 min, 100% B for re-equilibration. The flow rate was set to 0.3 mL/min. Mass spectrometric parameters were as follows: ion source gas 1, 30 psi; ion source gas 2, 80 psi; curtain gas, 40 psi; CAD gas, 8 arbitrary unit; ionspray voltage, −4500 V; temperature, 450°C. Precursor ion scan of m/z 305 Da with a scan range of m/z 480–1000 for Q1; EP, -10 V; CE, -53 V; CXP, -15 V in the negative ion mode was used to detect DGLA-containing phospholipids.

Polarity	Species (20:3-containing)	m/z
Positive ([M+H]+)	PC 36:3	784.5856
	PC 36:3+2	786.5982
	PC 36:3+3	787.6045
	PC 36:3+4	788.6107
	PC 36:3+5	789.6170
	PC 36:3+6	790.6233
	PC 36:3+7	791.6296
	PC 36:3+8	792.6358
	PC 38:3	812.6169
	PC 38:3+2	814.6295
	PC 38:3+3	815.6358
	PC 38:3+4	816.6420
	PC 38:3+5	817.6483
	PC 38:3+6	818.6546
	PC 38:3+7	819.6609
	PC 38:3+8	820.6671
Negative ([M-H]-)	PE 36:3	740.5230
	PE 36:3+2	742.5356
	PE 36:3+3	743.5419
	PE 36:3+4	744.5481
	PE 36:3+5	745.5544
	PE 36:3+6	746.5607
	PE 36:3+7	747.5670
	PE 36:3+8	748.5732
	PE 38:3	768.5543
	PE 38:3+2	770.5669
	PE 38:3+3	771.5732
	PE 38:3+4	772.5794
	PE 38:3+5	773.5857
	PE 38:3+6	774.5920
	PE 38:3+7	775.5983
	PE 38:3+8	776.6045
	PE O-36:4	724.5281
	PE O-36:4+2	726.5407
	PE O-36:4+3	727.5469
	PE O-36:4+4	728.5532
	PE O-36:4+5	729.5595
	PE O-36:4+6	730.5658
	PE O-36:4+7	731.5721
	PE O-36:4+8	732.5783
	PE O-38:5	752.5594
	PE O-38:5+2	754.5720
	PE O-38:5+3	755.5782
	PE O-38:5+4	756.5845
	PE O-38:5+5	757.5908
	PE O-38:5+6	758.5971
	PE O-38:5+7	759.6034
	PE O-38:5+8	760.6096
	PS 38:3	812.5442
	PS 38:3+2	814.5567
	PS 38:3+3	815.5630
	PS 38:3+4	816.5693
	PS 38:3+5	817.5755
	PS 38:3+6	818.5818
	PS 38:3+7	819.5881
	PS 38:3+8	820.5944
	PI 36:3	859.5337
	PI 36:3+2	861.5462
	PI 36:3+3	862.5525
	PI 36:3+4	863.5588
	PI 36:3+5	864.5650
	PI 36:3+6	865.5713
	PI 36:3+7	866.5776
	PI 36:3+8	867.5839
	PI 38:3	887.5650
	PI 38:3+2	889.5775
	PI 38:3+3	890.5838
	PI 38:3+4	891.5901
	PI 38:3+5	892.5963
	PI 38:3+6	893.6026
	PI 38:3+7	894.6089
	PI 38:3+8	895.6152

Polarity	Species (20:4-containing)	m/z
Positive ([M+H]+)	PC 36:4	782.5700
	PC 36:4+2	784.5825
	PC 36:4+3	785.5888
	PC 36:4+4	786.5951
	PC 36:4+5	787.6014
	PC 36:4+6	788.6076
	PC 36:4+7	789.6139
	PC 36:4+8	790.6202
	PC 38:4	810.6013
	PC 38:4+2	812.6138
	PC 38:4+3	813.6201
	PC 38:4+4	814.6264
	PC 38:4+5	815.6327
	PC 38:4+6	816.6389
	PC 38:4+7	817.6452
	PC 38:4+8	818.6515
Negative ([M-H]-)	PE 36:4	738.5074
	PE 36:4+2	740.5199
	PE 36:4+3	741.5262
	PE 36:4+4	742.5325
	PE 36:4+5	743.5388
	PE 36:4+6	744.5450
	PE 36:4+7	745.5513
	PE 36:4+8	746.5576
	PE 38:4	766.5387
	PE 38:4+2	768.5512
	PE 38:4+3	769.5575
	PE 38:4+4	770.5638
	PE 38:4+5	771.5701
	PE 38:4+6	772.5763
	PE 38:4+7	773.5826
	PE 38:4+8	774.5889
	PE O-36:5	722.5125
	PE O-36:5+2	724.5250
	PE O-36:5+3	725.5313
	PE O-36:5+4	726.5376
	PE O-36:5+5	727.5438
	PE O-36:5+6	728.5501
	PE O-36:5+7	729.5564
	PE O-36:5+8	730.5627
	PE O-36:5	752.5594
	PE O-36:5+2	754.5720
	PE O-36:5+3	755.5782
	PE O-36:5+4	756.5845
	PE O-36:5+5	757.5908
	PE O-36:5+6	758.5971
	PE O-36:5+7	759.6034
	PE O-36:5+8	760.6096
	PS 38:3	810.5285
	PS 38:3+2	812.5411
	PS 38:3+3	813.5473
	PS 38:3+4	814.5536
	PS 38:3+5	815.5599
	PS 38:3+6	816.5662
	PS 38:3+7	817.5724
	PS 38:3+8	818.5787
	PI 36:3	857.5180
	PI 36:3+2	859.5306
	PI 36:3+3	860.5368
	PI 36:3+4	861.5431
	PI 36:3+5	862.5494
	PI 36:3+6	863.5557
	PI 36:3+7	864.5619
	PI 36:3+8	865.5682
	PI 38:3	885.5493
	PI 38:3+2	887.5619
	PI 38:3+3	888.5681
	PI 38:3+4	889.5744
	PI 38:3+5	890.5807
	PI 38:3+6	891.5870
	PI 38:3+7	892.5932
	PI 38:3+8	893.5995

Table S1. Target analytes for LC-QTof-MS and MALDI-MS imaging

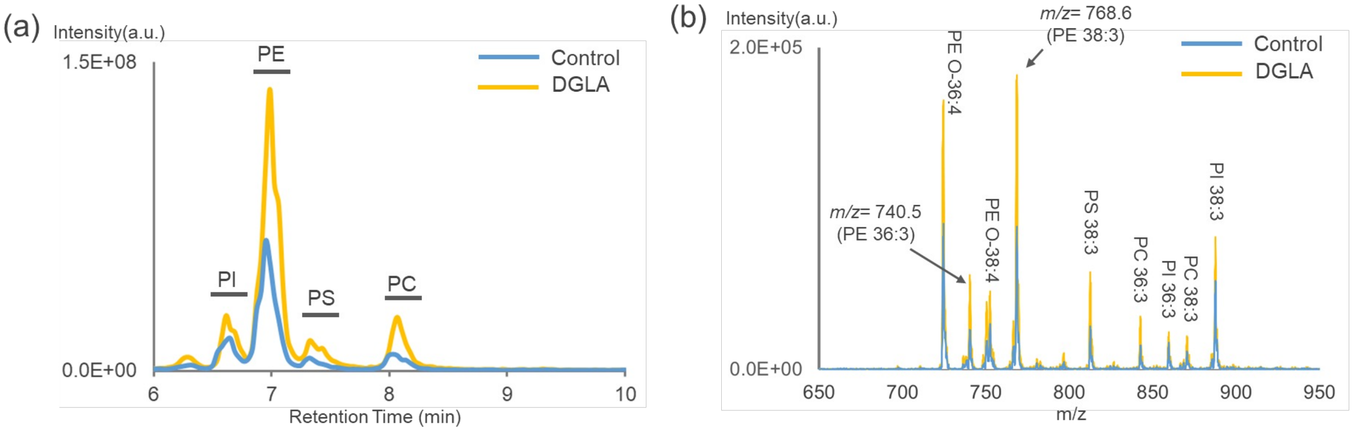

Figure S1. Analysis of DGLA-containing phospholipids in the mouse lung by LC-MS/MS.

(a) Precursor ion scan chromatograms for 20:3 fragment (*m/z*=305) of phospholipids from the mouse lung fed with control diet (blue) and mouse administered with DGLA (yellow) .

(b) Mass spectrums of phospholipid fraction (6-9 min). Precursor ions that produced the 20:3 fragment (m/z 305) in (a) were assigned to DGLA-containing molecular species based on MS/MS

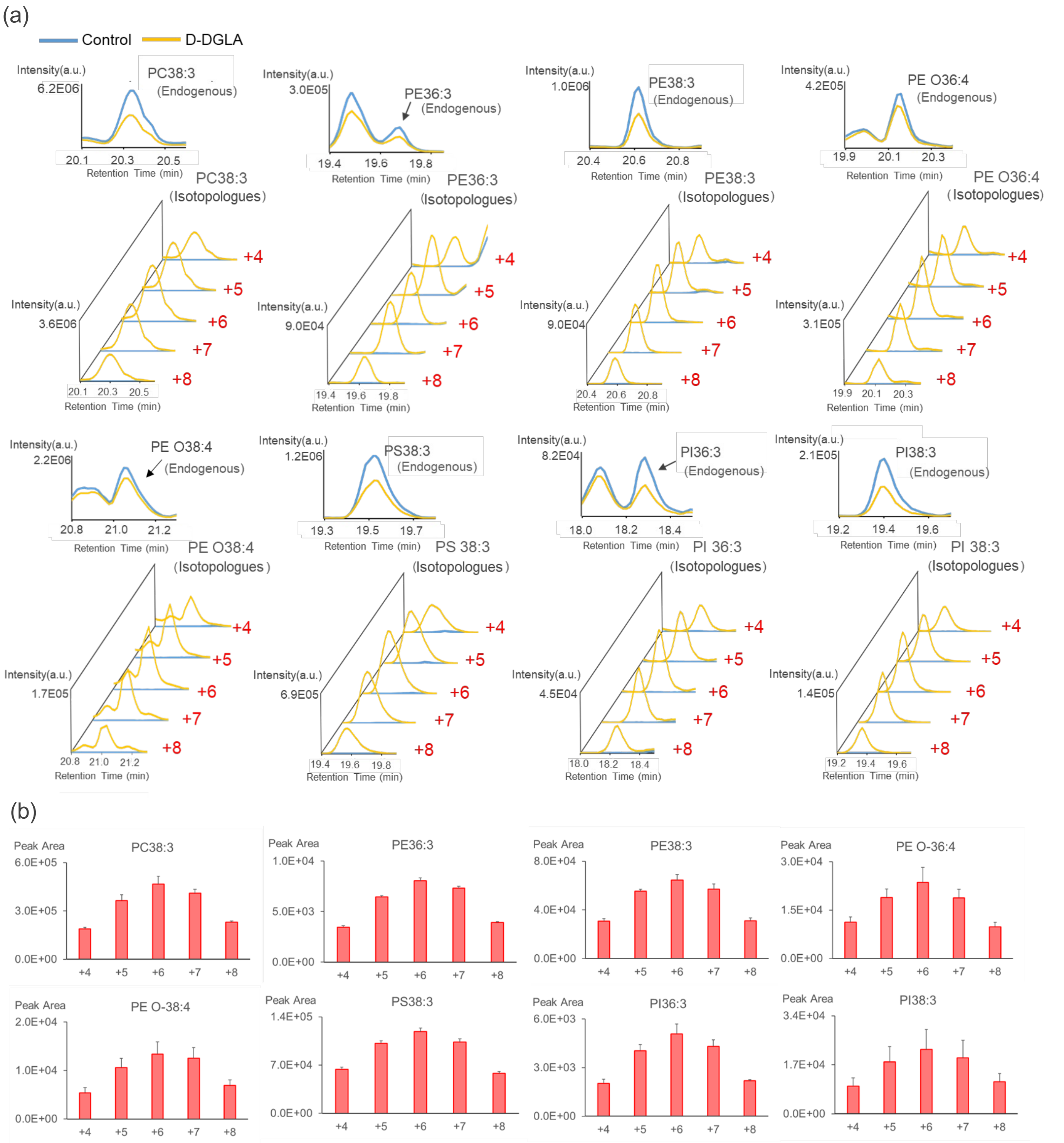

Figure S2. **Analysis of D-DGLA-containing-phospholipids in the mouse lung administered with D-DGLA by LC-QTof-MS.**

(a) Extracted ion chromatograms for endogenous eight phospholipid species (upper) and the corresponding phospholipid (PL) isotopologues (lower) in the lung of mice fed with control diet (blue) and mice administered with D-DGLA (yellow). Note that first-eluting peaks of endogenous PL36:3 are expected be composition isomers.

(b) Peak areas for PL36:3 and PL38:3 isotopologues in the lung of D-DGLA administered mice. Data are presented as mean+S.D. (n=3 biological replicates; individual mice). “+X” indicates the nominal mass shift (Da) of each D-DGLA isotopologue relative to unlabeled DGLA (defined as +0), corresponding to the number of incorporated deuterium atoms.

Analysis result for PC36:3 is shown in Figure 3a, b.

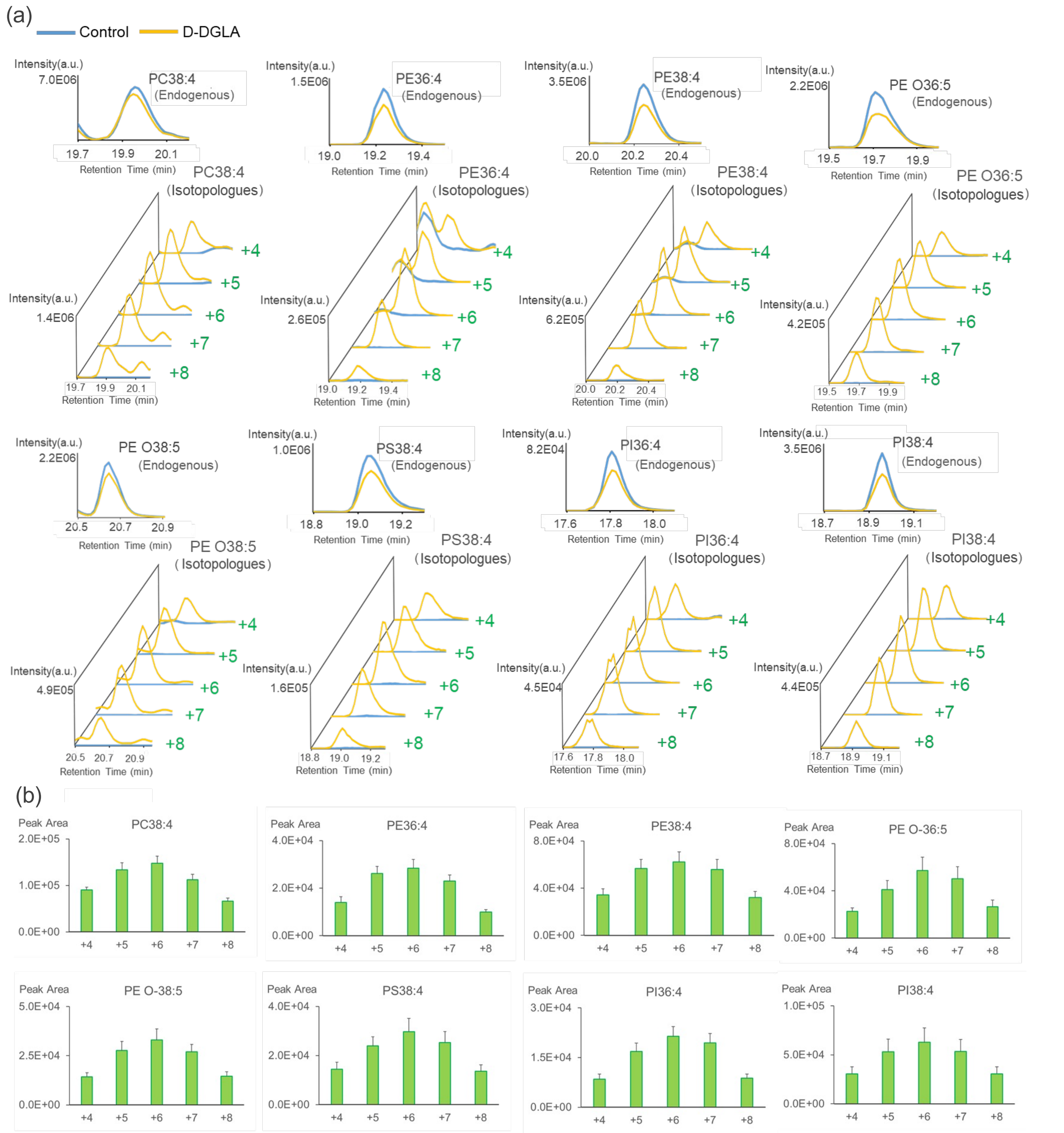

Figure S3. Analysis of D-ARA-containing-phospholipids in the mouse lung administered with D-DGLA by LC-QTof-MS.

(a) Extracted ion chromatograms for endogenous eight phospholipid species (upper) and the corresponding phospholipids (PL) isotopologues (lower) in the lung of mice fed with control diet (blue) and mice administered with D-DGLA (yellow).

(b) Peak areas for PL36:4 and PL38:4 isotopologues in the lung of D-DGLA administered mice. Data are presented as mean+S.D. (n=3 biological replicates; individual mice). “+X” indicates the nominal mass shift (Da) of each D-ARA isotopologue relative to unlabeled ARA (defined as +0), corresponding to the number of incorporated deuterium atoms.

Analysis result for PC36:4 is shown in Figure 3c, d.

Species		Substitution rate (%)[a]
DGLA containing	PC36:3	80.7
	PC38:3	78.0
	PE36:3	81.4
	PE38:3	81.1
	PE O-36:4	68.7
	PE O-38:4	65.1
	PS38:3	78.0
	PI36:3	77.7
	PI38:3	80.7
ARA containing	PC36:4	50.8
	PC38:4	41.7
	PE36:4	49.9
	PE38:4	46.9
	PE O-36:5	51.5
	PE O-38:5	41.9
	PS38:4	49.2
	PI36:4	49.4
	PI38:4	77.7

Table S2. Substitution rate of DGLA and ARA-containing phospholipids

[a] *Substitution rates were calculated as follows. Substitution rate (%) = [Intensity_isotopologues / (Intensity_isotopologues + Intensity_endogenous)] × 100, where Intensity_ isotopologues is the summed peak intensity of major isotopologues (+4 to +8),*
and Intensity_endogenous is the peak intensity of the endogenous phospholipid.

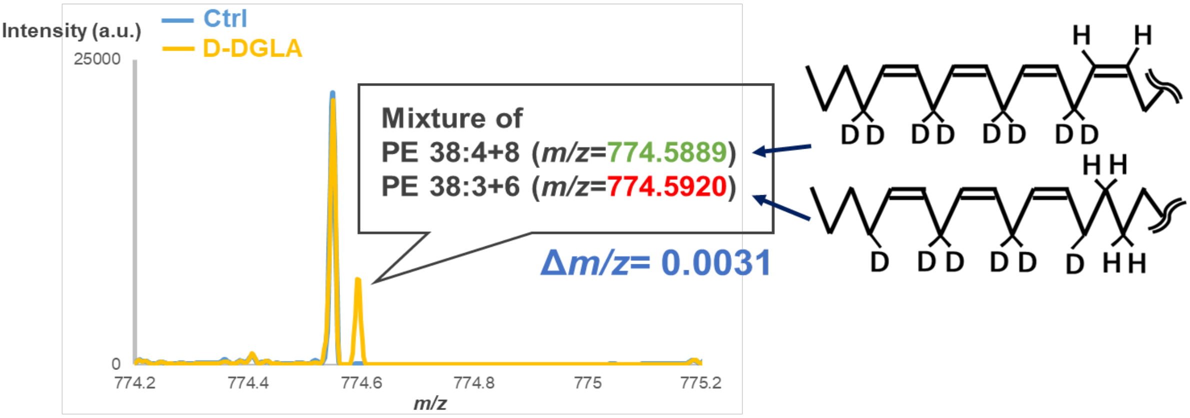

Figure S4. An example of overlapping *m/z* values between D-DGLA- and D-ARA-containing phospholipids.

D-DGLA can be converted to D-ARA, which is also incorporated into phospholipids. The *m/z* values of phospholipids containing +6 D-DGLA isotopologue and those containing +8 D-ARA isotopologue are nearly identical, differing by only 0.0031. Therefore, it is difficult to detect them separately in MS imaging. Here we show the example of PE, but the same relationship is also true for other phospholipid classes.

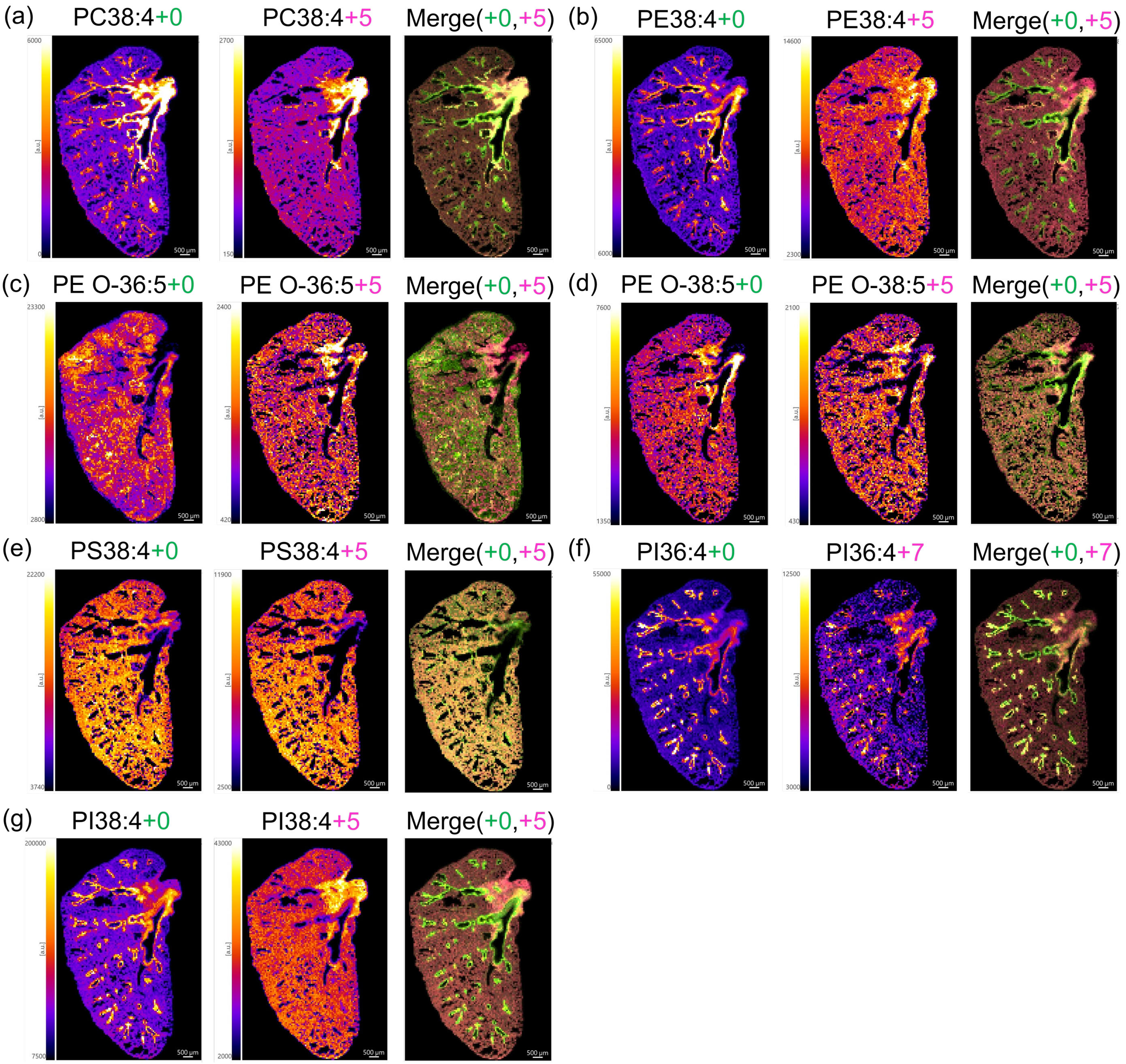

Figure S5. Visualization of D-ARA-containing phospholipids in the mouse lung in comparison with phospholipids containing endogenous ARA.

(a-g) Distribution of endogenous ARA-containing phospholipid (PL+0) is shown on the left, D-ARA-containing phospholipid (PL+X) is in the middle, and the merged image is on the right. Isotopologues with the lowest background shown in Figure 5 were selected. The isotopologues selected are; (a) PC38:4+0 and +5, (b) PE38:4+0 and +5, (c) PE O-36:5+0 and +5, (d) PE O-38:4+0 and +5, (e) PS38:4+0 and +5, (f) PI36:4+0 and +7, (g) PI38:4+0 and +5. Spatial resolution=70 µm. . "+X" denotes the nominal mass shift (Da) of each D-ARA isotopologue relative to unlabeled ARA (defined as +0), corresponding to the number of incorporated deuterium atoms. The MS imaging experiment was independently performed twice with similar results, and representative images are shown. In the merged images, signals from endogenous lipids are shown in green, and those from D-ARA-containing lipids are shown in magenta.

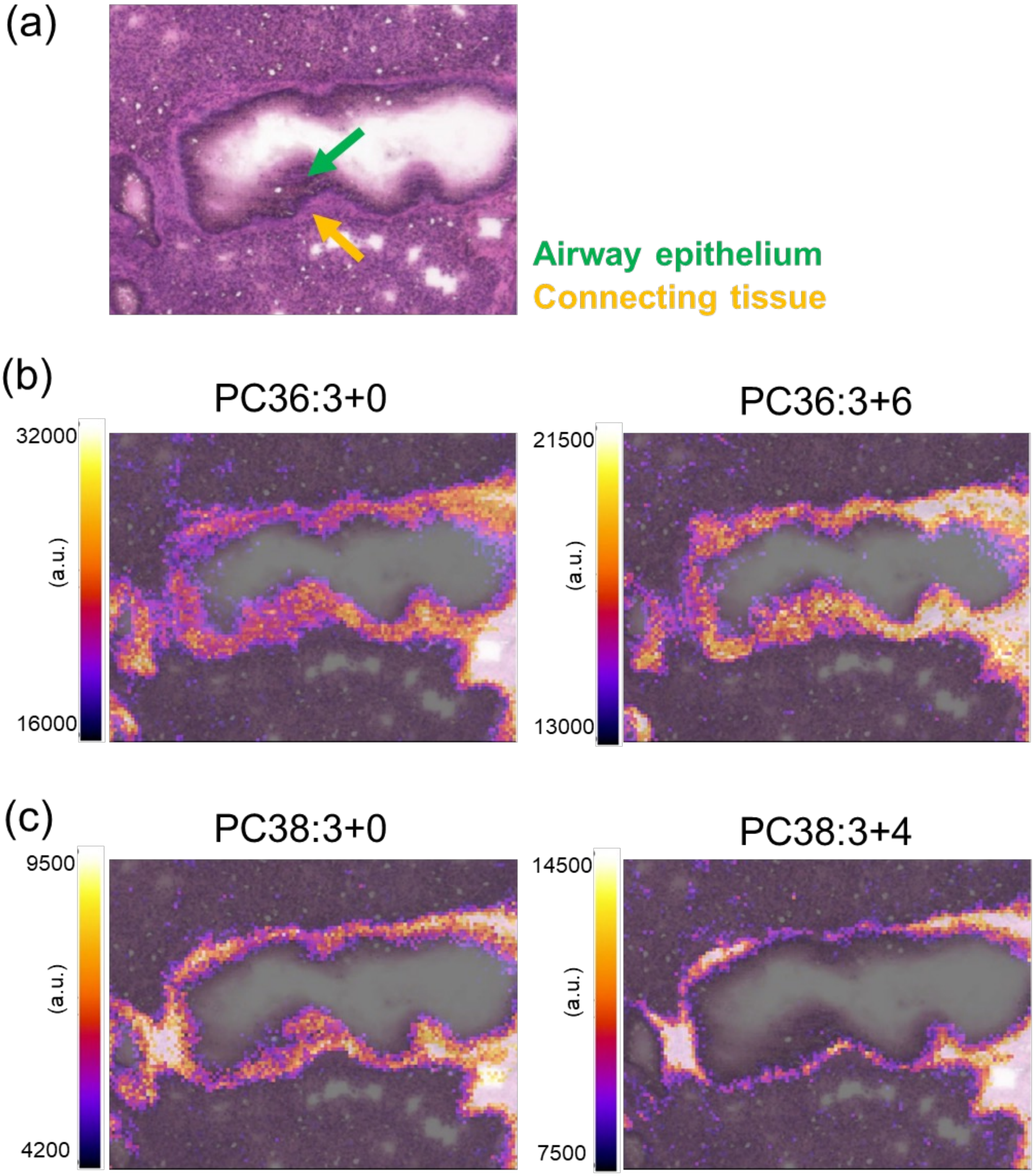

Figure S6. D-DGLA is differently incorporated into PC36:3 and PC38:3.
(a) HE staining of airway regions. (b, c) High-resolution images (10 μm spatial resolution) of PC36:3 (38:3) and their D-DGLA-containing counterparts in airway regions of D-DGLA-administered mice. (b) Overlay of PC36:3 (+0 and +6) and HE images. (c) Overlay of PC38:3 (+0 and +4) and HE images. Note that D-DGLA is primarily incorporated into PC 36:3 in the epithelium, but in the underlying connective tissue, it is incorporated into both PC36:3 and PC38:3.

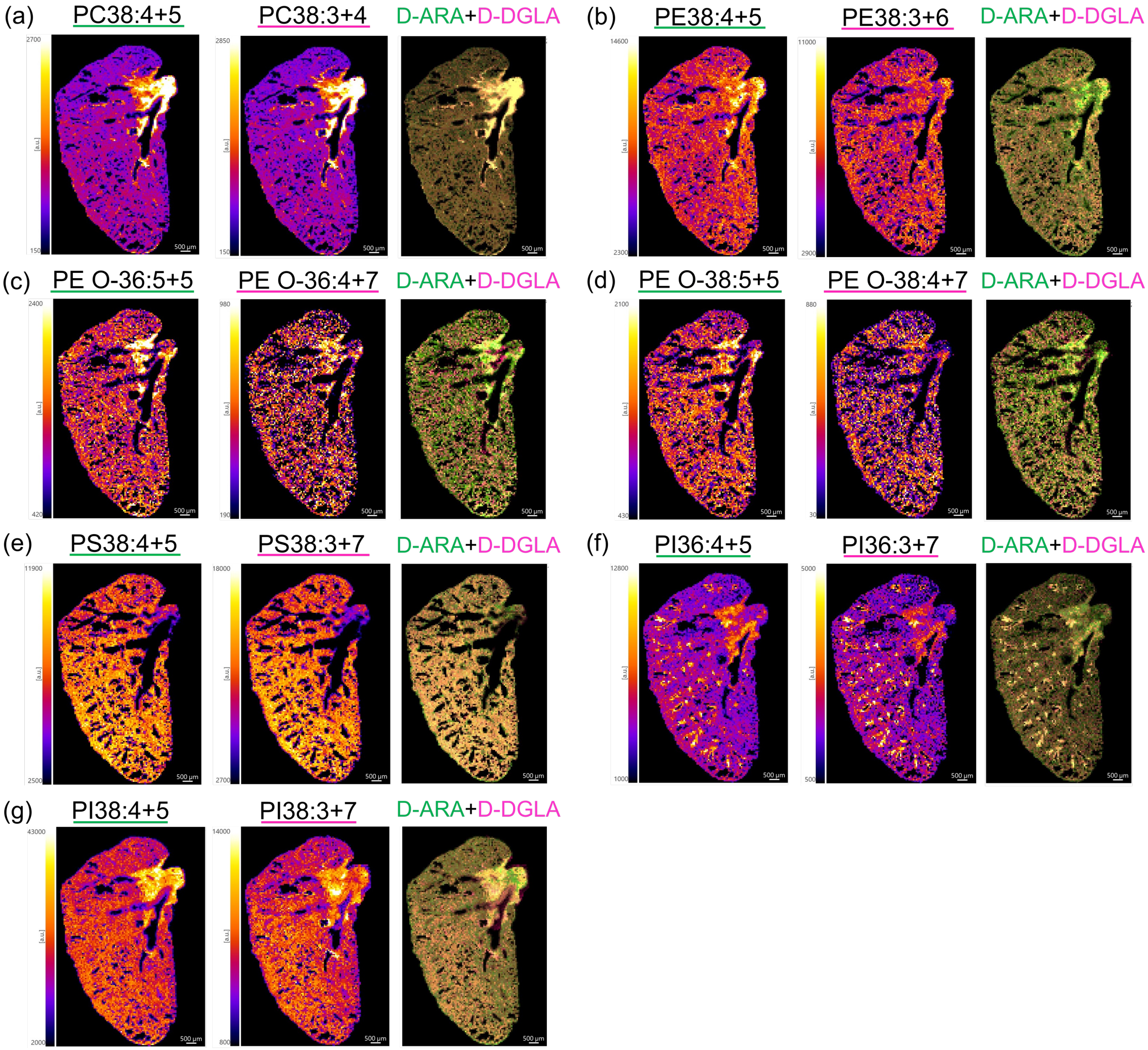

Figure S7. Visualization of D-DGLA-containing phospholipids in the mouse lung in comparison with D-ARA-containing phospholipids.

(a-g) Distribution of D-ARA- (left) and D-DGLA-containing phospholipids (middle) of each species. Merged images are the right. Isotopologues with the lowest background shown in Figure 5 were selected. The pairs used are as follows; (a) PC38:4+5 and PC38:3+4, (b) PE38:4+5 and PE38:3+6, (c) PS38:4+5 and PS38:3+7, (d) PI36:4+7 and PI36:3+7, (e) PI38:4+5 and PI38:3+7. Spatial resolution=70 μm. Note that structurally similar phospholipid species exhibited nearly identical spatial distributions. . "+X" denotes the nominal mass shift (Da) of each D-DGLA/ARA isotopologue relative to unlabeled DGLA/ARA (defined as +0), corresponding to the number of incorporated deuterium atoms. The MS imaging experiment was independently performed twice with similar results, and representative images are shown. In the merged images, signals from D-DGLA-containing lipids are shown in green, and those from D-ARA-containing lipids are shown in magenta.

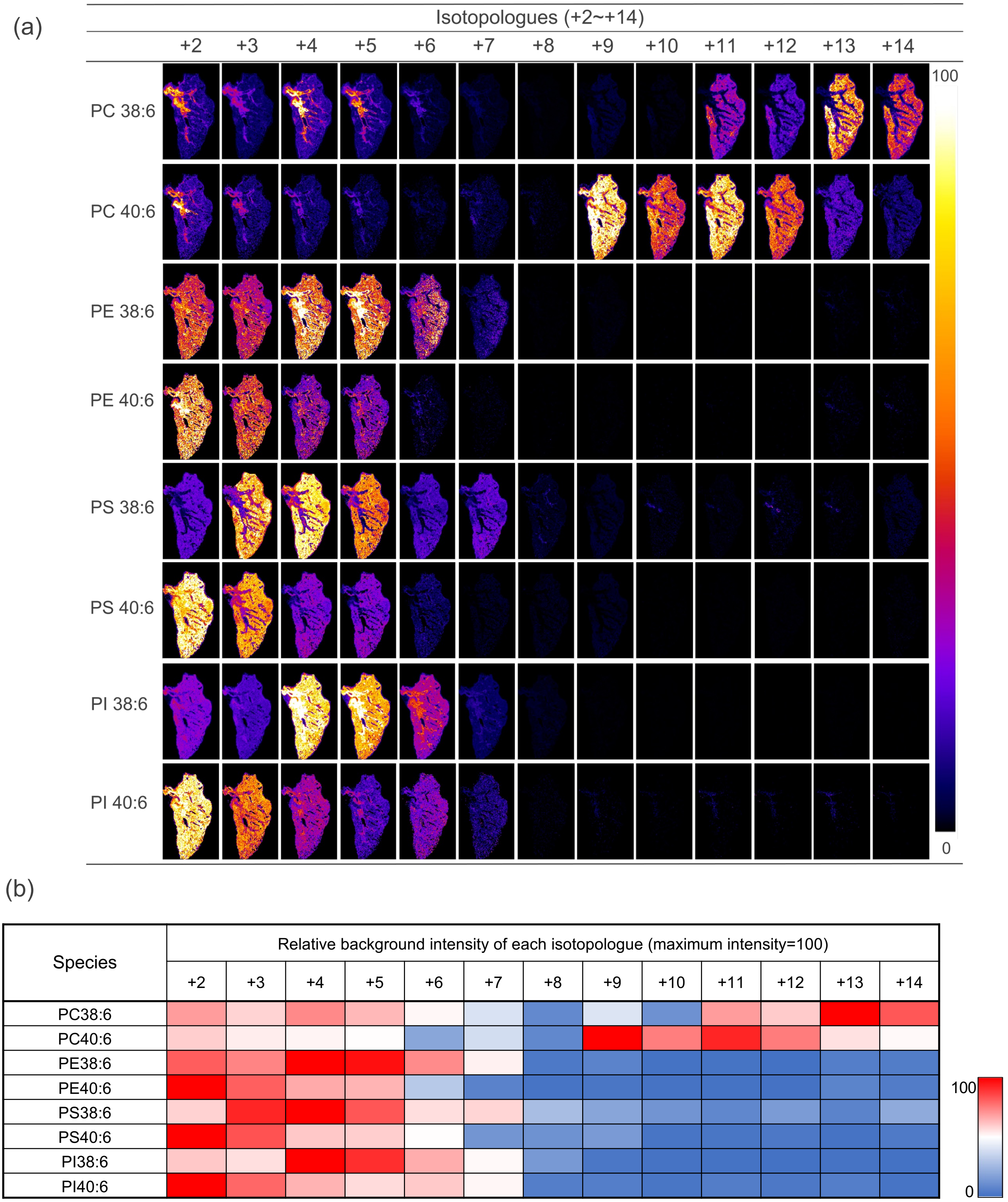

Figure S8. **Comparison of relative intensities of *m/z* values putatively corresponding to DHA isotopologues in mouse lung by MALDI-MS imaging**

(a) MS imaging of *m/z* values putatively corresponding to phospholipids containing DHA isotopologues(+2~+14).

(b) Heatmap summarizing the average intensities in (a). For each molecular species, the Isotopologue with the maximum intensity was normalized to 100. Note that background signals were higher for +2 to +4, even though these isotopologues were used in earlier studies.

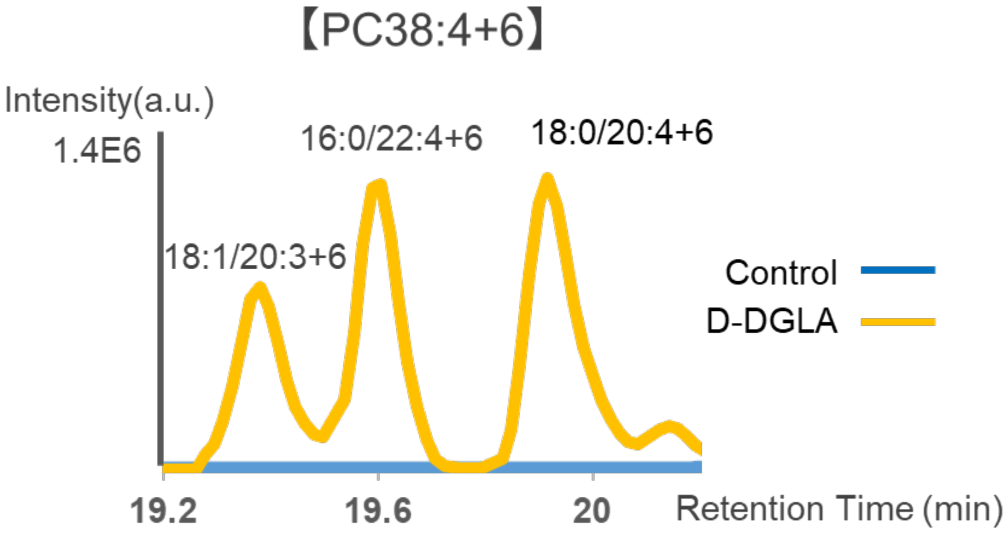

Figure S9. An example in which multiple D-DGLA-derived phospholipids with the same molecular formula were detected.

D-DGLA administration led to the detection of structural isomers of PC 38:4 containing DGLA, ARA, or adrenic acid (22:4n-6). Although these isomers can be separated by LC-QTof-MS, they cannot be distinguished in MSI using MS1 alone. More detailed localization of each molecular species could be achieved by using MS2 or ion mobility.
